# Self-harm hospitalizations and neighbourhood level material and social deprivation in Canada: an ecological study

**DOI:** 10.1186/s12888-024-06316-8

**Published:** 2024-11-29

**Authors:** Li Liu, Nathaniel J. Pollock, Gisèle Contreras, Yuan Xu, Wendy Thompson

**Affiliations:** Centre for Surveillance and Applied Research, Public Health Agency of Canada, 785 Carling Avenue, Ottawa, ON K1S 5H4 Canada

**Keywords:** Self-harm, Suicide, Health care, Social deprivation, Material deprivation, Socio-economic status, Neighbourhood

## Abstract

**Background:**

Socio-economic status is associated with self-harm at the individual and area level. In Canada, there is limited evidence on the relationship between area-level markers of socio-economic status and self-harm. The objective of this study was to assess the impact of small area-level material and social deprivation on rates of hospitalization due to self-harm.

**Methods:**

Administrative data on hospitalizations from two databases in Canada (Discharge Abstract Database and Ontario Mental Health Reporting System) for the period April 1, 2015 to March 31, 2022, were analyzed. Rates of self-harm hospitalization and percentage of repeated admissions were estimated across quintiles of material and social deprivation. Rate ratios were computed to evaluate disparities. The rates were stratified by sex and age group; the percentage of repeated admissions were stratified by sex.

**Results:**

In total, the study included 109,398 hospitalizations due to self-harm. Populations in more deprived areas had higher rates of self-harm hospitalizations than those in less deprived areas. The rate ratios for people who lived in the most deprived areas over the least deprived areas were 1.48 (95% CI: 1.38–1.58) and 1.71 (95% CI: 1.60–1.82) for material and social deprivation, separately. The largest disparity was among people aged 25–44 years for material and 45–64 years for social deprivation. Percentages of repeated self-harm hospitalizations were significantly higher in more deprived areas compared to less deprived areas for social deprivation, and among males but not females for material deprivation.

**Conclusions:**

Both material and social deprivation were associated with self-harm hospitalization and repeated admissions; the disparity varied by subgroup and the deprivation components. This study demonstrated a need to consider interventions at the neighbourhood level and address both community and population-level conditions of social and material need.

**Supplementary Information:**

The online version contains supplementary material available at 10.1186/s12888-024-06316-8.

## Introduction

Self-harm, including deliberate acts of self-injury and self-poisoning, is one of the strongest risk factors for suicide [1, 2]. The etiology of self-harm is complex and involves biological, psychosocial, and environmental factors [3]. In particular, socio-economic context has a strong influence on suicide and self-harm, though less is known about the effects on repeat hospitalization [4].

Previous studies found that deprivation at the individual level greatly increased the rate of self-harm [5–7]. Evidence also suggests that contextual factors that operate at the ecological level may also influence self-harm. A systematic review of studies from Europe reported significant associations between area-level material deprivation and self-harm even after adjusting for covariates [8]. A more recent study from the United Kingdon found that populations in the most materially deprived areas accounted for almost half of self-harm hospital presentations across five general hospitals [9]. A Danish cohort study found that people in affluent neighbourhoods had lower incidence rates of self-harm compared to those in deprived neighbourhoods [10]. An Irish study found that deprivation is the strongest independent area-level predictor of self-harm after adjusting for other explanatory variables [11]. Similarly, the rate of self-harm was positively associated with deprivation domains of employment, crime, education, health and income in Northern Ireland, and that self-harm rates were more than four times higher in the most deprived area [12].

Relatively few studies have investigated the relationship between self-harm and social dimensions of deprivation. Social fragmentation measured by Condon’s index had been used to represent social deprivation content. Social fragmentation is a composite measure derived from census data on proportions of population turnover, single-person households, households privately renting, and non-married adults [13–15]. One study conducted in Manchester, England found that the composite measure of social fragmentation was not associated with self-harm [15]. Another study found that social fragmentation was only associated with increased self-harm in rural districts in Ireland [16]. Some studies found that the association between self-harm and social fragmentation is stronger among females compared to males [17, 18].

To date, relatively few studies have examined area-level deprivation and self-harm in the Canadian context [19–24], and the findings are not consistent. A study in Quebec found that while socio-economic inequalities in suicide attempts persisted over time between 1990 and 2005, the greatest disparities were observed among people aged 24–44 years and diminished with increasing age [20]. Another study based on emergency department presentations by 12–17 year-olds in Ontario reported that the incidence rate for self-harm was inversely correlated to neighbourhood income [24]. A pan-Canadian study examined the association between hospitalizations for self-harm and area-level income inequality, and did not find statistically significant results [23]. Finally, another Canadian study examined the association between suicide-related behaviours (an outcome that included self-harm emergency department visits, hospitalizations, and deaths) and neighbourhood deprivation, and found neighbourhood deprivation to be associated with higher odds of suicide-related behaviours, with some evidence of dose–response effect where increased suicide-related behaviours risk were seen in higher levels of deprivation [22].

History of self-harm is not associated only with suicide but also with future repeated self-harm [25, 26]. In pooled estimations reported in a meta-analysis with over 170 international studies, the overall rate of non-fatal repetition of self-harm within 1 year was 16.3% [1]. Risk factors for repeat self-harm include sex (female), number of previous self-harm episodes, self-cutting as means of injury, mental disorders, alcohol abuse/dependence, drug abuse/dependence, and living alone [4]. The role of area-level factors on repeat self-harm, however, remains understudied. One study from England published in 2006 found that an index of deprivation was associated with self-harm presenting to emergency departments but this association was not observed for repeat self-harm; only area-based proportion of individuals who were of White ethnicity was associated with repetition of self-harm [27].

It is important to understand how these contextual factors contribute to the risk of self-harm. The objectives of this study were to estimate the rate of self-harm hospitalizations from 2015 to 2022 and the percentage of patients with repeated self-harm hospital admissions over the course of the study period by area-level material and social deprivation, and investigate the disparities across levels of deprivation.

## Methods

### Data sources

Data in this study cover hospitalizations due to self-harm for patients discharged between the fiscal years 2015 to 2021 (April 1, 2015 to March 31, 2022). The data were obtained from the Canadian Institute of Health Information (CIHI)’s Discharge Abstract Database (DAD) and Ontario Mental Health Reporting System (OMHRS). The data in DAD used in this study include acute care facilities from all provinces and territories in Canada except Quebec. OMHRS captured self-harm hospitalizations in Ontario for patients who received mental health services but were not recorded in DAD. The study covers 71.5% of the population in Canada and 92.6% of the population in Canada outside of Quebec.

Self-harm cases were identified using the International Statistical Classification of Diseases and Related Health Problems, 10th Revision, Canada (ICD-10-CA). For DAD, we searched 25 diagnostic fields for any hospitalization codes related to self-harm (X60-X84 and Y87.0). OMHRS does not use ICD-10 codes. Since most patients were admitted to hospital for self-harm via the emergency department, we linked the data from OMHRS with the National Ambulatory Care Reporting System (NACRS). Hospitalizations recorded in OMHRS with prior emergency department visits within 7 days due to intentional self-harm (based on ICD-10 codes) were identified as self-harm hospitalizations [28]. Data were accessed through CIHI’s secure access environment.

For data on area-level deprivation, we used the Material and Social Deprivation Index developed by the Institut national de santé publique du Québec (INSPQ) [29]. The index is based on the dissemination area (DA), which is the smallest standard geographic area for national census data, with an average population of 400–700 people. The material deprivation component includes measures of the proportion of the population without a high school diploma or equivalent, the employment to population ratio, and the average individual income for the population aged 15 years or over. The social deprivation component is a composite measure of the proportion of the population aged 15 or over living alone, the proportion of the population who are separated, divorced or widowed, and the proportion of single-parent families [29].

For each component, the measures were combined into a deprivation index through principal component analysis that generated a factor score. The DAs were ranked on the basis of the index and then divided into quintiles. Quintile 1 represents the population living in the most privileged (least deprived) DA and quintile 5 represents the most deprived DA. The material and social deprivation index based on the data from the 2016 census were used in this study [30].

### Statistical analysis

We calculated the age-standardized and age-specific rates of self-harm hospitalization per 100,000 population across the quintiles of material and social deprivation. In the rate calculations, the denominators were the 2016 population estimates from Statistics Canada and the standard population used for age-standardized rates was the 2011 Canadian Census population. We computed rate ratios across the quintiles of the deprivation index by negative binomial regression models, which mitigate the impact of overdispersion, using the 1st quintile group, the least deprived area, as the reference group. The models were adjusted for sex and age group, when these variables were not stratified. In order to account for the impact of the COVID-19 pandemic, the models adjusted for the pandemic period using April 2020 as the cutoff point.

Additionally, we conducted stratified analyses by age group using the following population groups: children (0–14 years), youth (15–19 years), emerging adults (20–24 years), young adults (25–44 years), middle-aged adults (45–64 years), and older adults (65 years or over). The analyses were also stratified by sex. Patients with sex other than female or male were not analyzed separately in sex-stratified analyses due to small counts, but were included in the overall and other stratified analyses. For sensitivity analysis, we analysed the data for the pre-pandemic period and during the pandemic separately.

If a patient had more than one self-harm hospitalizations when living in a deprivation area during the study period, we classified this patient as having repeated self-harm hospitalizations at this deprivation level. We estimated prevalence of repeated self-harm hospitalizations among patients with self-harm hospitalizations across deprivation levels, and computed the unadjusted prevalence ratios and the ratios adjusting sex, age group and province/territory using negative binomial regression models.

Since the study was based on de‐identified data and conducted under the surveillance mandate of the Public Health Agency of Canada, no ethics approval from an Institutional Review Board was sought.

## Results

We identified a total of 121,278 hospitalizations due to self-harm from April 1, 2015 to March 31, 2022; this study included 109,398 hospitalizations that were linked with the deprivation index. The linked hospitalizations were from 80,313 unique patients, including 50,546 females, 29,746 males, and the remaining 21 with sex not female or male or sex unknown. There were 892 hospitalizations that did not have a unique personal identifier, and we excluded them only for the counts of unique patients and the analyses on repeated admissions. The frequency and distribution of self-harm hospitalizations are shown in Table 1. Females accounted for more than two-thirds of all self-harm hospitalizations; females aged 0–14 years, had more than 8 times the number of hospitalizations compared to males in the same age group. Deprived areas had more hospitalizations due to self-harm than privileged areas, for both females and males. Patients from the most materially and socially deprived areas (the 5th quintile) accounted for 25.7% and 28.7% of the total number of self-harm hospitalizations, respectively; while the corresponding percentage from the least deprived areas (the 1st quintile) were 15.5% and 16.1%.

**Table 1:** Number and percentage of self-harm hospitalizations, fiscal years 2015 – 2021, Canada excluding Quebec

	**Category**	**All sexes** **N (%)**	**Female** **N (%)**	**Male** **N (%)**
**Overall**		109,398 (100.0)	70,729 (100.0)	38,575 (100.0)
**Age group, years**	0–14	6893 (6.3)	6146 (8.7)	733 (1.9)
	15–19	20,860 (19.1)	16,384 (23.2)	4449 (11.5)
	20–24	14,963 (13.7)	10,004 (14.1)	4926 (12.8)
	25–44	33,413 (30.5)	19,441 (27.5)	13,954 (36.2)
	45–64	25,593 (23.4)	14,749 (20.9)	10,842 (28.1)
	65 +	7675 (7.0)	4005 (5.7)	3670 (9.5)
**Material** **deprivation**	Q1	16,921 (15.5)	11,055 (15.6)	5848 (15.2)
	Q2	19,604 (17.9)	13,002 (18.4)	6585 (17.1)
	Q3	21,994 (20.1)	14,119 (20.0)	7860 (20.4)
	Q4	22,785 (20.8)	14,514 (20.5)	8246 (21.4)
	Q5	28,067 (25.7)	18,027 (25.5)	10,021 (26.0)
**Social** **deprivation**	Q1	17,565 (16.1)	11,459 (16.2)	6097 (15.8)
	Q2	18,568 (17.0)	12,201 (17.3)	6353 (16.5)
	Q3	19,202 (17.6)	12,464 (17.6)	6728 (17.4)
	Q4	22,644 (20.7)	14,741 (20.8)	7891 (20.5)
	Q5	31,392 (28.7)	19,852 (28.1)	11,491 (29.8)

*Abbreviation*: *Q1-Q5* Quintiles of deprivation index (Q1: least deprived area; Q5: most deprived area)

**Fig. 1:**
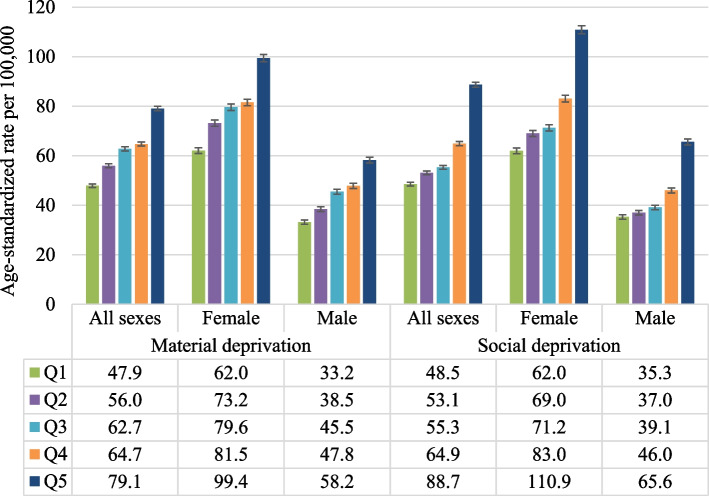
Age-standardized rate per 100,000 population of self-harm hospitalizations across material and social deprivation, by sex, fiscal years 2015 – 2021, Canada excluding Quebec. Q1-Q5: quintiles of deprivation index (Q1: least deprived area; Q5: most deprived area)

Figure 1 shows the age-standardized rates of self-harm hospitalization across quintiles of material and social deprivation overall, and by sex. For both females and males, the rate of self-harm hospitalizations tended to increase with deprivation. For material deprivation, females had a rate of 99.4 (95% CI: 97.9–100.8) per 100,000 population for the most deprived area, compared to 62.0 (95% CI: 60.9–63.2) per 100,000 in the least deprived area, while the corresponding numbers for males were 58.2 (95% CI: 57.1–59.3) per 100,000 and 33.2 (95% CI: 32.4–34.1) per 100,000. Similarly, for social deprivation, females had a rate of 110.9 (95% CI: 109.3–112.4) per 100,000 for the most deprived area compared to 62.0 (95% CI: 60.8–63.1) per 100,000 in the least deprived area; the corresponding rates for males were 65.6 (95% CI: 64.3–66.8) per 100,000 and 35.3 (95% CI: 34.3–36.2) per 100,000. Table 2 presents the rate ratios across quintiles of the deprivation index. After adjusting for covariates, the rates in the most deprived area were 1.48 (95% CI: 1.38–1.58) times higher than the least deprived area for the material components, and 1.71 (95% CI: 1.60–1.82) times for the social components. The rate ratios were similar for females and males for all ages combined.

**Table 2:** Rate ratio of self-harm hospitalizations across the quintile of material and social deprivation using Q1, the least deprived area, as reference, overall and by sex, fiscal years 2015 – 2021, Canada excluding Quebec

Quintile	Material deprivation		Social deprivation	
	**Unadjusted rate ratio** **(95% CI)**	**Adjusted rate ratio** **(95% CI)**	**Unadjusted rate ratio** **(95% CI)**	**Adjusted rate ratio** **(95% CI)**
**Overall**				
**Q2**	1.15 (0.99, 1.34)	**1.16 (1.08, 1.24)**	1.13 (0.97, 1.31)	**1.08 (1.01, 1.15)**
**Q3**	**1.24 (1.06, 1.44)**	**1.27 (1.19, 1.36)**	1.15 (0.99, 1.33)	**1.11 (1.04, 1.18)**
**Q4**	**1.20 (1.03, 1.39)**	**1.25 (1.17, 1.34)**	**1.30 (1.12, 1.51)**	**1.26 (1.18, 1.34)**
**Q5**	**1.43 (1.23, 1.66)**	**1.48 (1.38, 1.58)**	**1.70 (1.46, 1.97)**	**1.71 (1.60, 1.82)**
**Female**				
**Q2**	1.16 (0.99, 1.37)	**1.18 (1.11, 1.26)**	1.16 (0.99, 1.37)	**1.12 (1.06, 1.18)**
**Q3**	**1.22 (1.03, 1.43)**	**1.24 (1.17, 1.32)**	1.17 (1.00, 1.38)	**1.12 (1.06, 1.18)**
**Q4**	1.18 (1.00, 1.39)	**1.22 (1.15, 1.30)**	**1.32 (1.12, 1.56)**	**1.26 (1.20, 1.33)**
**Q5**	**1.41 (1.19, 1.66)**	**1.44 (1.36, 1.53)**	**1.68 (1.43, 1.98)**	**1.70 (1.61, 1.79)**
**Male**				
**Q2**	1.16 (0.99, 1.35)	**1.12 (1.05, 1.21)**	1.05 (0.90, 1.23)	1.04 (0.97, 1.11)
**Q3**	**1.31 (1.12, 1.53)**	**1.28 (1.19, 1.37)**	1.09 (0.93, 1.28)	**1.08 (1.01, 1.15)**
**Q4**	**1.28 (1.09, 1.50)**	**1.22 (1.14, 1.31)**	**1.21 (1.04, 1.42)**	**1.22 (1.14, 1.30)**
**Q5**	**1.52 (1.30, 1.78)**	**1.43 (1.33, 1.54)**	**1.65 (1.41, 1.93)**	**1.65 (1.55, 1.77)**

Adjusted rate ratios adjusted for sex, age group and pandemic period. Statistically significant rate ratios are bolded (*p*-value < 0.05)

*Abbreviation*: *Q1-Q5* Quintiles of deprivation index (Q1: least deprived area; Q5: most deprived area), *CI* Confidence interval

**Fig. 2:**
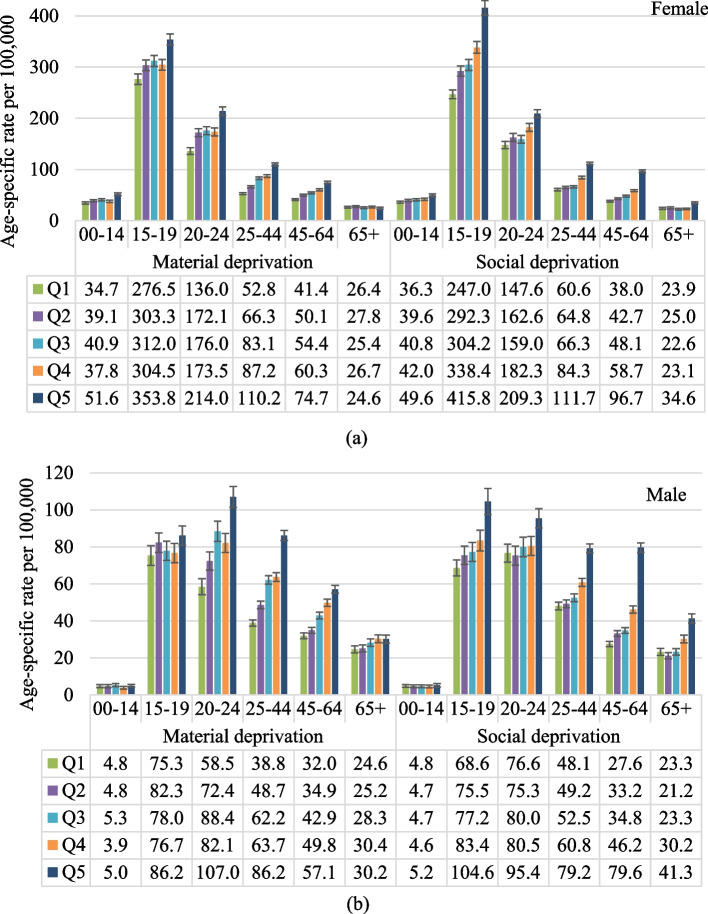
Age-specific rate per 100,000 population of self-harm hospitalizations across material and social deprivation index, by age group for (**a**) females and (**b**) males, fiscal years 2015 – 2021, Canada excluding Quebec. Q1-Q5: quintiles of deprivation index (Q1: least deprived area; Q5: most deprived area)

Figure 2 shows age specific rate of self-harm hospitalizations across deprivation level by age group. For females, the most deprived areas had the highest rates across the quintiles of both material and social deprivation, except for the rate of those aged 65 years and older for material deprivation, which was close to or slightly lower than the rates in other areas. The highest rates for females were observed in the age group 15–19 years for both material and social deprivation. For males, across the deprivation level, the rates in the most deprived area were the highest among young and middle-aged adults (i.e., those aged 20–64 years) for material deprivation, and among all age groups for social deprivation. Table 3 and supplementary Table S1 presents rate ratios by age group. Disparities in the rate of self-harm hospitalizations across deprivation level varied by age group. For material deprivation, 25–44 years had the highest rate ratios, with nearly double the rates in the most deprived areas compared to the least deprived area for both females with rate ratio of 2.04 (95% CI: 1.79–2.33) and males 2.11 (95% CI: 1.88–2.27); no significant rate ratios were observed for males aged 0–14 and 15–19 years, but were significant for most of the deprivation levels for females in the same age groups. For social deprivation, the age group 45–64 years had the highest rate ratios, 2.48 (95% CI: 2.20–2.79) for females and 2.78 (95% CI: 2.45–3.15) for males, in the most deprived area compared to the least deprived area, but was not significant for young males aged 0–14 years. For sensitivity analysis, we conducted the same analyses using the data before and during pandemic period separately shown in the supplementary Tables S2 and S3. We found the similar patterns before and during pandemic period for most of population subgroups.

**Table 3:** Unadjusted rate ratio of self-harm hospitalizations across the quintile of material and social deprivation using Q1, the least deprived area, as reference for females and males, by age group, fiscal years 2015 – 2021, Canada excluding Quebec

Quintile	Unadjusted rate ratio (95% CI)			
	**Female**		**Male**	
	**Material deprivation**	**Social deprivation**	**Material deprivation**	**Social deprivation**
**10–14 years**				
**Q2**	1.15 (0.98, 1.34)	1.14 (0.97, 1.34)	1.03 (0.80, 1.32)	1.00 (0.78, 1.29)
**Q3**	**1.20 (1.03, 1.41)**	1.16 (0.98, 1.36)	1.12 (0.88, 1.44)	1.00 (0.78, 1.29)
**Q4**	1.08 (0.92, 1.26)	1.17 (0.99, 1.37)	0.82 (0.64, 1.06)	0.99 (0.77, 1.28)
**Q5**	**1.48 (1.27, 1.74)**	**1.37 (1.17, 1.62)**	1.04 (0.81, 1.33)	1.18 (0.92, 1.53)
15–19 years				
**Q2**	1.09 (0.98, 1.22)	**1.21 (1.11, 1.31)**	1.08 (0.95, 1.24)	1.12 (1.00, 1.26)
**Q3**	1.12 (1.00, 1.25)	**1.25 (1.15, 1.35)**	1.06 (0.93, 1.21)	**1.14 (1.01, 1.28)**
**Q4**	1.06 (0.95, 1.19)	**1.38 (1.27, 1.50)**	1.00 (0.88, 1.15)	**1.23 (1.09, 1.38)**
**Q5**	**1.23 (1.10, 1.37)**	**1.69 (1.56, 1.84)**	1.11 (0.97, 1.27)	**1.53 (1.36, 1.72)**
**20–24 years**				
**Q2**	**1.25 (1.11, 1.41)**	**1.13 (1.01, 1.28)**	**1.23 (1.10, 1.37)**	1.00 (0.87, 1.15)
**Q3**	**1.27 (1.13, 1.43)**	1.10 (0.97, 1.23)	**1.50 (1.34, 1.68)**	1.05 (0.92, 1.21)
**Q4**	**1.22 (1.08, 1.37)**	**1.25 (1.11, 1.41)**	**1.37 (1.22, 1.53)**	1.04 (0.90, 1.19)
**Q5**	**1.52 (1.35, 1.71)**	**1.45 (1.29, 1.64)**	**1.78 (1.59, 2.00)**	**1.24 (1.08, 1.42)**
**25–44 years**				
**Q2**	**1.29 (1.13, 1.48)**	1.13 (0.99, 1.28)	**1.25 (1.11, 1.41)**	1.07 (0.94, 1.22)
**Q3**	**1.62 (1.42, 1.84)**	1.13 (1.00, 1.29)	**1.59 (1.41, 1.79)**	1.12 (0.98, 1.28)
**Q4**	**1.62 (1.42, 1.85)**	**1.42 (1.25, 1.61)**	**1.58 (1.40, 1.77)**	**1.29 (1.13, 1.47)**
**Q5**	**2.04 (1.79, 2.33)**	**1.99 (1.75, 2.25)**	**2.11 (1.88, 2.37)**	**1.77 (1.55, 2.02)**
**45–64 years**				
**Q2**	**1.26 (1.06, 1.50)**	**1.13 (1.01, 1.28)**	1.15 (0.95, 1.40)	**1.19 (1.05, 1.35)**
**Q3**	**1.35 (1.13, 1.61)**	**1.25 (1.11, 1.41)**	**1.38 (1.14, 1.68)**	**1.23 (1.09, 1.40)**
**Q4**	**1.42 (1.19, 1.69)**	**1.51 (1.34, 1.70)**	**1.52 (1.25, 1.84)**	**1.62 (1.43, 1.83)**
**Q5**	**1.71 (1.43, 2.03)**	**2.48 (2.20, 2.79)**	**1.67 (1.37, 2.03)**	**2.78 (2.45, 3.15)**
**65 years and over**				
**Q2**	1.10 (0.95, 1.26)	1.01 (0.90, 1.15)	1.06 (0.89, 1.26)	0.88 (0.77, 1.01)
**Q3**	1.01 (0.87, 1.16)	0.91 (0.80, 1.03)	1.17 (0.98, 1.39)	0.97 (0.84, 1.11)
**Q4**	1.06 (0.92, 1.23)	0.95 (0.84, 1.08)	**1.23 (1.03, 1.46)**	**1.26 (1.10, 1.44)**
**Q5**	0.91 (0.79, 1.05)	**1.41 (1.24, 1.59)**	1.18 (1.00, 1.41)	**1.71 (1.50, 1.96)**

Statistically significant rate ratios are bolded (*p*-value < 0.05)

*Abbreviation*: *Q1-Q5* Quintiles of deprivation index (Q1: least deprived area; Q5: most deprived area), *CI* Confidence interval

Table 4 shows the percentage of patients with repeated self-harm hospital admissions during the study period by deprivation levels. The percentage of patients with repeated hospitalization due to self-harm was significantly associated with social deprivation for both females and males. Overall, 18.6% (95% CI: 18.1–19.1) of patients in the most socially deprived areas had repeated self-harm hospitalizations, compared to 13.8% (95% CI: 13.2–14.4) of those who lived in the least socially deprived area. The prevalence ratio of repeated self-harm admissions was 1.29 (95% CI: 1.23, 1.36) for people living in the most social deprived area over the least deprived area after adjusting sex, age group and province/territory. For material deprivation, a significant association was observed among males but not females. The percentages are 13.6% (95% CI: 12.6–14.6) and 15.9% (95% CI: 15.1–16.7) for males who lived in the least and most materially deprived areas, respectively.

**Table 4:** Prevalence and prevalence ratio of repeated self-harm hospitalizations among people with self-harm hospitalizations across the quintile of material and social deprivation, fiscal years 2015 – 2021, Canada excluding Quebec

Quintile	Material deprivation			Social deprivation		
	**Prevalence, % (95% CI)**	**Unadjusted prevalence ratio** **(95% CI)**	**Adjusted prevalence ratio** **(95% CI)**	**Prevalence, % (95% CI)**	**Unadjusted prevalence ratio** **(95% CI)**	**Adjusted prevalence ratio** **(95% CI)**
**Overall**						
Q1	15.0 (14.4, 15.6)	(Ref)	(Ref)	13.8 (13.2, 14.4)	(Ref)	(Ref)
Q2	14.8 (14.3, 15.4)	0.99 (0.93, 1.05)	0.95 (0.90, 1.01)	13.4 (12.9, 13.9)	0.97 (0.91, 1.03)	0.98 (0.92, 1.04)
Q3	15.3 (14.7, 15.8)	1.02 (0.96, 1.08)	0.95 (0.90, 1.01)	14.5 (13.9, 15.0)	1.05 (0.99, 1.11)	**1.07 (1.01, 1.14)**
Q4	15.8 (15.3, 16.3)	1.05 (0.99, 1.11)	0.97 (0.91, 1.02)	15.8 (15.3, 16.4)	**1.15 (1.08, 1.22)**	**1.14 (1.08, 1.21)**
Q5	16.5 (16.0, 16.9)	**1.09 (1.04, 1.16)**	1.02 (0.97, 1.08)	18.6 (18.1, 19.1)	**1.35 (1.28, 1.42)**	**1.29 (1.23, 1.36)**
**Female**						
Q1	15.8 (15.1, 16.6)	(Ref)	(Ref)	14.5 (13.8, 15.2)	(Ref)	(Ref)
Q2	15.9 (15.2, 16.6)	1.01 (0.94, 1.08)	0.97 (0.90, 1.04)	14.2 (13.5, 14.9)	0.98 (0.91, 1.05)	0.99 (0.92, 1.06)
Q3	16.0 (15.3, 16.7)	1.01 (0.94, 1.09)	0.95 (0.89, 1.02)	15.3 (14.6, 16.0)	1.05 (0.98, 1.13)	1.07 (0.99, 1.15)
Q4	16.5 (15.8, 17.2)	1.04 (0.97, 1.12)	0.96 (0.89, 1.03)	16.4 (15.7, 17.0)	**1.13 (1.05, 1.21)**	**1.12 (1.04, 1.20)**
Q5	16.7 (16.1, 17.4)	1.06 (0.99, 1.13)	0.99 (0.93, 1.06)	19.4 (18.8, 20.0)	**1.34 (1.25, 1.43)**	**1.28 (1.20, 1.37)**
**Male**						
Q1	13.6 (12.6, 14.6)	(Ref)	(Ref)	12.5 (11.6, 13.4)	(Ref)	(Ref)
Q2	12.8 (12.0, 13.7)	0.94 (0.85, 1.05)	0.91 (0.82, 1.01)	11.9 (11.1, 12.8)	0.95 (0.85, 1.06)	0.95 (0.85, 1.06)
Q3	14.0 (13.1, 14.8)	1.03 (0.93, 1.14)	0.95 (0.86, 1.05)	13.1 (12.2, 14.0)	1.05 (0.94, 1.16)	1.07 (0.96, 1.19)
Q4	14.6 (13.8, 15.5)	1.07 (0.97, 1.19)	0.97 (0.88, 1.08)	14.9 (14.1, 15.8)	**1.19 (1.08, 1.32)**	**1.18 (1.07, 1.31)**
Q5	15.9 (15.1, 16.7)	**1.17 (1.07, 1.29)**	1.07 (0.98, 1.18)	17.2 (16.4, 18.0)	**1.37 (1.25, 1.50)**	**1.30 (1.19, 1.43)**

Adjusted prevalence ratio adjusted for sex, age group and province/territory; Bold font represents prevalence ratio significantly differs from 1 at *p* < 0.05

*Abbreviation*: *CI* Confidence interval, *Q1-Q5* Quintiles of deprivation index (Q1: least deprived area; Q5: most deprived area), *CI* Confidence interval, *Ref*: Reference group

## Discussion

This study used administrative health data from Canada to investigate the relationship between hospitalizations due to self-harm and area-level material and social deprivation. People who lived in the most deprived areas had significantly higher rates of self-harm hospitalizations compared to those who lived in the most privileged areas, even after adjusting for covariates. This association was observed for females and males and for most age groups. The results also showed that deprivation levels were associated with repeated self-harm hospitalizations, though the associations varied by the type of deprivation and sex.

Our results of area-level association are aligned with other research that indicates greater likelihood and rates of self-harm, for people living in areas with higher area-level deprivation [8–12]. From a systematic review of studies in Europe, 22 of 27 studies found a strong positive association and three found some evidence of association between area-level deprivation and suicidal behaviours including deliberate self-harm [8]. A Canadian study indicated that elevated suicide risk was not limited to people with low socio-economic status (particularly those who were not legally married, who were unemployed and who earned low income), but also extended to those who lived in the areas with higher area-level deprivation [19].

People who live in more deprived areas may have lower level of social support, greater overall stress, higher risk for mental health issues such as depression [31] and psychotic disorders [32], less access to mental health services as well as other resources, which increased the risk of self-harm. These factors may also contribute to less effective follow-up care and informal support for people who are discharged from hospital following a suicide attempt if they live in socially and materially deprived areas. This in turn may increase the likelihood of repeated hospitalizations.

We observed that both females and males aged 25–44 years old living in the most materially deprived areas had about twice the rate of self-harm hospitalizations compared to those in the least deprived areas. In contrast, older adults ages 45–64 years who lived in the most socially deprived areas had two to three times the rate of self-harm hospitalizations compared to those in the least deprived areas. This suggests a strong association between deprivation and self-harm risk in these population subgroups. The different impacts on age groups may be due to varying types of stressors and life circumstances associated with each form of deprivation at different life stages. Material deprivation may be related more to economic instability and related stressors for younger adults, whereas social deprivation might relate to social isolation and reduced support networks for older adults.

Consistent with other studies [26, 33], this study also found higher percentages of patients in the most deprived neighbourhoods that had repeat hospitalizations for self-harm over the study period. As repeat hospitalizations raise questions about appropriateness of services and follow-up care, and gaps in the health care system, these results are concerning. Mechanisms underlying the effect of material deprivation and social deprivation on self-harm and repeat self-harm remain poorly understood. Highly materially deprived areas may be characterized by less investment for programmes in education, health care, and mental health resources, which may lead to high rates of self-harm. Socially deprived areas may increase a lack of perceived belonging, a declining sense of neighbourhood identification, and experiences of social rejection which may increase the risk of self-harm [34, 35]. More work is needed to understand the pathways that explain the relationship between deprivation and self-harm and warrants further investigation.

Additionally, among the most deprived, there was a greater percentage of repeat self-harm hospitalizations for social deprivation compared to material deprivation (18.6% versus 16.5%). Our measures of deprivation were based on composite score and precluded examining the effect of individual components, but it is possible that some of the components may play a more important role in the association with repeat self-harm than others. For example, living alone (a component of the social deprivation index in this study) is a known risk factor for repeat self-harm, with those who live alone up to 3 times more likely to repeat self-harm [4]. It is possible that living alone results in lower social support from others that is needed in seeking and obtaining the help and resources, placing these individuals at greater risk for repeat episodes of self-harm requiring hospitalization. Future studies should examine individual components of social and material deprivation to determine if differences are present in their association with repeated self-harm.

### Strengths and limitations

This study used administrative data on self-harm hospitalizations that covered most areas and populations in Canada. The data sources extended to hospital discharges recorded in OMHRS in addition to DAD − the data source that most Canadian studies have used − which provide more complete estimates of self-harm hospitalizations for Ontario. We also used integrated deprivation indices containing material and social components separately to represent neighbourhood-level socio-economic status. Moreover, our study examined self-harm and deprivation based on small geographical unit area to ensure a relatively homogenous population size and minimize the variation of area-level measures within the area.

However, this study has several limitations. Because of the availability of the data, we could not adjust for more demographic characteristics beyond sex and age. Factors such as employment, education, income, mental disorder, marital status, and racialized or Indigenous identity may impact the association between self-harm hospitalizations and neighbourhood deprivation, which had been found for the association between suicide and deprivation [19]. Other factors, such as rurality and geographic location may influence the association between deprivation and self-harm, which we are not able to adjust for due to data availability. A previous study had found high rates of self-harm hospitalizations in the most remote areas of Canada [36]. Moreover, due to a lack of data access, our study did not include hospitalizations from Quebec. Though the estimates do not represent the whole national level, our study still covers 76% of the population in Canada. This national study only focuses on self-harm hospitalizations and does not include emergency department (ED) visits from the data of NACRS because only a few jurisdictions (Ontario, Alberta and Yukon) mandatorily report data to the NACRS. Moreover, a previous study found that only a small portion of self-harm ED visits were captured in NACRS [37]. Due to data availability, we used age group 0–14 years. The rates may be underestimated for age group 10–14 years because few self-harm cases occurred among children under 10 years old. In addition, the ICD-10 codes used to identify self-harm cases did not permit distinction between cases with or without suicidal intent.

## Conclusions

Rates of self-harm hospitalizations are strongly associated with area-level material and social deprivation. People who lived in more deprived areas had higher rates of self-harm hospitalizations than those who lived in less deprived areas. The associations were observed in both females and males but varied by age group. The largest disparity in the rates of self-harm hospitalizations across area-level deprivation was among people aged 25–44 years for material deprivation and aged 45–64 years for social deprivation. People who lived in socially deprived areas and males in materially deprived areas were more likely to have repeat self-harm hospitalizations. These results suggest that deprivation at the area level may play a role in ecological risks for self-harm and underscore the need for leveraging local public health interventions as a key strategy in suicide prevention. Investments in social services, mental health care, and community support, and reducing poverty in deprived areas may help mitigate the higher risk of self-harm and improve overall well-being.

## Supplementary Information

Supplementary Material 1.

## Data Availability

Individual level data were accessed through agreements between Public Health Agency of Canada and Canadian Institution of Health Information (CIHI). Data can be requested through CIHI https://www.cihi.ca/en/access-data-and-reports/data-holdings/make-a-data-request.

## References

[1] Carroll R, Metcalfe C, Gunnell D. Hospital presenting self-harm and risk of fatal and non-fatal repetition: systematic review and meta-analysis. PLoS ONE. 2014;9(2):e89944. 10.1371/journal.pone.0089944. 24587141 10.1371/journal.pone.0089944 PMC3938547

[2] Owens D, Horrocks J, House A. Fatal and non-fatal repetition of self-harm. Systematic review Br J Psychiatry. 2002;181:193–9. 10.1192/bjp.181.3.193. 12204922 10.1192/bjp.181.3.193

[3] Turecki G, Brent DA. Suicide and suicidal behaviour. Lancet. 2016;387(10024):1227–39. 10.1016/S0140-6736(15)00234-2. 26385066 10.1016/S0140-6736(15)00234-2 PMC5319859

[4] Larkin C, Di Blasi Z, Arensman E. Risk factors for repetition of self-harm: a systematic review of prospective hospital-based studies. PLoS ONE. 2014;9(1):e84282. 10.1371/journal.pone.0084282. 24465400 10.1371/journal.pone.0084282 PMC3896350

[5] Ayton A, Rasool H, Cottrell D. Deliberate self-harm in children and adolescents: association with social deprivation. Eur Child Adolesc Psychiatry. 2003;12(6):303–7. 10.1007/s00787-003-0344-0. 14689263 10.1007/s00787-003-0344-0

[6] Knipe DPP, Newton-Howes G, Chan LF, Kapur N. Suicide and self-harm. Lancet. 2022;379(9834):2373–82. 10.1016/S0140-6736(12)60322-5.

[7] Nilsson AF, Nordentoft M. How family income is associated with suicidal and violent behaviour in young adults. The Lancet Public Health. 2018;3(10):E463–4. 10.1016/S2468-2667(18)30181-6. 30314587 10.1016/S2468-2667(18)30181-6

[8] Cairns JM, Graham E, Bambra C. Area-level socioeconomic disadvantage and suicidal behaviour in Europe: A systematic review. Soc Sci Med. 2017;192:102–11. 10.1016/j.socscimed.2017.09.034. 28965001 10.1016/j.socscimed.2017.09.034

[9] Geulayov G, Casey D, Bale E, et al. Socio-economic disparities in patients who present to hospital for self-harm: patients’ characteristics and problems in the Multicentre Study of Self-harm in England. J Affect Disord. 2022;318:238–45. 10.1016/j.jad.2022.08.106. 36055531 10.1016/j.jad.2022.08.106

[10] Ejlskov L, Antonsen S, Wulff JN, et al. Multilevel interactions between family and neighbourhood socioeconomic indices in childhood and later risks of self-harm and violent criminality in Denmark: a national cohort study. Lancet Public Health. 2023;8(2):e99–108. 10.1016/S2468-2667(22)00292-4. 36709062 10.1016/S2468-2667(22)00292-4 PMC9896147

[11] O’Farrell IB, Corcoran P, Perry IJ. Characteristics of small areas with high rates of hospital-treated self-harm: deprived, fragmented and urban or just close to hospital? A national registry study. J Epidemiol Community Health. 2015;69(2):162–7. 10.1136/jech-2014-204587. 25320248 10.1136/jech-2014-204587

[12] Griffin E, Bonner B, Dillon CB, et al. The association between self-harm and area-level characteristics in Northern Ireland: an ecological study. Eur J Public Health. 2019;29(5):948–53. 10.1093/eurpub/ckz021. 30851111 10.1093/eurpub/ckz021

[13] Congdon P. Suicide and Parasuicide in London: A Small-area Study. Urban Studies, 1996. 33(1). 10.1080/00420989650012194.

[14] Stjarne MK, Ponce de Leon A, Hallqvist J. Contextual effects of social fragmentation and material deprivation on risk of myocardial infarction--results from the Stockholm Heart Epidemiology Program (SHEEP). Int J Epidemiol. 2004;33(4):732–41. 10.1093/ije/dyh087. 10.1093/ije/dyh087 15155706

[15] Lin CY, Bickley H, Clements C, et al. Spatial patterning and correlates of self-harm in Manchester. England Epidemiol Psychiatr Sci. 2019;29:e72. 10.1017/S2045796019000696. 31739808 10.1017/S2045796019000696 PMC8061130

[16] Corcoran P, Arensman E, Perry IJ. The area-level association between hospital-treated deliberate self-harm, deprivation and social fragmentation in Ireland. J Epidemiol Community Health. 2007;61(12):1050–5. 10.1136/jech.2006.055855. 18000126 10.1136/jech.2006.055855 PMC2465671

[17] Harriss L, Hawton K. Deliberate self-harm in rural and urban regions: a comparative study of prevalence and patient characteristics. Soc Sci Med. 2011;73(2):274–81. 10.1016/j.socscimed.2011.05.011. 21684647 10.1016/j.socscimed.2011.05.011

[18] Congdon P. Assessing the impact of socioeconomic variables on small area variations in suicide outcomes in England. Int J Environ Res Public Health. 2012;10(1):158–77. 10.3390/ijerph10010158. 23271304 10.3390/ijerph10010158 PMC3564135

[19] Burrows S, Auger N, Gamache P, et al. Influence of social and material individual and area deprivation on suicide mortality among 2.7 million Canadians: a prospective study. BMC Public Health, 2011. 11: p. 577. 10.1186/1471-2458-11-577. 10.1186/1471-2458-11-577 PMC3154174 21771330

[20] Burrows S, Auger N, Roy M, Alix C. Socio-economic inequalities in suicide attempts and suicide mortality in Quebec, Canada, 1990–2005. Public Health. 2010;124(2):78–85. 10.1016/j.puhe.2010.01.008. 20181370 10.1016/j.puhe.2010.01.008

[21] Zandy M, Zhang LR, Kao D, et al. Area-based socioeconomic disparities in mortality due to unintentional injury and youth suicide in British Columbia, 2009–2013. Health Promot Chronic Dis Prev Can. 2019;39(2):35–44. 10.24095/hpcdp.39.2.01. 30767853 10.24095/hpcdp.39.2.01 PMC6394817

[22] Azra KK, Nielsen A, Kim C, et al. Investigating suicide related behaviours across sexual orientation and neighbourhood deprivation levels: A cohort study using linked health administrative data. PLoS ONE. 2023;18(3):e0282910. 10.1371/journal.pone.0282910. 36989270 10.1371/journal.pone.0282910 PMC10058080

[23] Benny C, Siddiqi A, Pabayo R. Income inequality and “hospitalisations of despair” in Canada: a study on longitudinal, population-based data. J Epidemiol Community Health. 2023. 10.1136/jech-2023-220900. 37739771 10.1136/jech-2023-220900

[24] Bethell J, Bondy SJ, Lou WY, et al. Emergency department presentations for self-harm among Ontario youth. Can J Public Health. 2013;104(2):e124–30. 10.1007/BF03405675. 23618204 10.1007/BF03405675 PMC6974110

[25] Zahl DL, Hawton K. Repetition of deliberate self-harm and subsequent suicide risk: long-term follow-up study of 11,583 patients. Br J Psychiatry. 2004;185:70–5. 10.1192/bjp.185.1.70. 15231558 10.1192/bjp.185.1.70

[26] Hunter J, Maunder R, Kurdyak P, et al. Mental health follow-up after deliberate self-harm and risk for repeat self-harm and death. Psychiatry Res. 2018;259:333–9. 10.1016/j.psychres.2017.09.029. 29120839 10.1016/j.psychres.2017.09.029

[27] Johnston A, Cooper J, Webb R, Kapur N. Individual- and area-level predictors of self-harm repetition. Br J Psychiatry. 2006;189:416–21. 10.1192/bjp.bp.105.018085. 17077431 10.1192/bjp.bp.105.018085

[28] Canadian Institute for Health Information (CIHI). Self-harm hospitalizations [indicator]. 2023. https://www.cihi.ca/en/indicators/self-harm-hospitalizations. Accessed 10 March 2024.

[29] Gamache P, Hamel D, Blaser C. Bureau d’information et d’études en santé des populations Material and social deprivation index: A summary. 2019. http://www.inspq.qc.ca/en/publications/2639. Accessed 10 March 2024.

[30] Institut national de santé publique du Québec (INSPQ). Index of material and social deprivation compiled by the Bureau d'information et d'études en santé des populations (BIESP) from 1991, 1996, 2001, 2006, 2011 and 2016 Canadian Census data. 2023. https://www.inspq.qc.ca/en/deprivation/material-and-social-deprivation-index. Accessed 10 March 2024.

[31] O’Donoghue B, Roche ELane A. Neighbourhood level social deprivation and the risk of psychotic disorders: a systematic review. Soc Psychiatry Psychiatr Epidemiol. 2016;51(7):941–50. 10.1007/s00127-016-1233-4. 27178430 10.1007/s00127-016-1233-4

[32] Blair A, Ross NA, Gariepy G, Schmitz N. How do neighborhoods affect depression outcomes? A realist review and a call for the examination of causal pathways. Soc Psychiatry Psychiatr Epidemiol. 2014;49(6):873–87. 10.1007/s00127-013-0810-z. 24414031 10.1007/s00127-013-0810-z

[33] de la Torre-Luque A, Pemau A, Ayad-Ahmed W, et al. Risk of suicide attempt repetition after an index attempt: A systematic review and meta-analysis. Gen Hosp Psychiatry. 2023;81:51–6. 10.1016/j.genhosppsych.2023.01.007. 36805332 10.1016/j.genhosppsych.2023.01.007

[34] Cawley RPE, Touhey J, Sheehy K, Taylor PJ. What is the relationship between rejection and self-harm or suicidality in adulthood? J Affect Disord. 2019;242:123–34. 10.1016/j.jad.2018.08.082. 30173060 10.1016/j.jad.2018.08.082

[35] McIntyre JEA, Latham C, Mullholland H, Haines-Delmont A, Saini P, Taylor PJ. Does neighbourhood identification buffer against the effects of socioeconomic disadvantage on self-harm? J Affect Disord. 2021;294:857–63. 10.1016/j.jad.2021.07.103. 34375213 10.1016/j.jad.2021.07.103

[36] Mahinpey N, Pollock NJ, Liu L, et al. Self‑harm and rurality in Canada: an analysis of hospitalization data from 2015 to 2019. Social Psychiatry and Psychiatric Epidemiology, 2023. 10.1007/s00127-023-02463-7. 10.1007/s00127-023-02463-7 PMC10081931 37029322

[37] Johnson D, Skinner R, Cappelli M, et al. Self-Inflicted Injury-Canadian Hospitals Injury Reporting and Prevention Program (CHIRPP-SI): a new surveillance tool for detecting self-inflicted injury events in emergency departments. Can J Public Health. 2019;110(2):244–52. 10.17269/s41997-018-0139-1. 30311176 10.17269/s41997-018-0139-1 PMC6964488

