# Supplementary material for: Self-harm hospitalizations and neighbourhood level material and social deprivation in Canada: an ecological study
Source: BMC Psychiatry. 2024 Nov 29;24:859. doi: 10.1186/s12888-024-06316-8 (PMC11606135; doi:10.1186/s12888-024-06316-8)
Supplement: Supplementary file 1 — Supplementary Material 1. [file 12888_2024_6316_MOESM1_ESM.docx]

**Self-harm hospitalizations and neighbourhood level material and social deprivation in Canada: An ecological study**

**Supplementary material**

Table S1 Adjusted rate ratio of self-harm hospitalizations across the quintile of material and social deprivation using Q1, the least deprived area, as reference for females and males, by age group, fiscal years 2015 – 2021, Canada excluding Quebec

| **Quintile** | **Adjusted rate ratio (95% CI)** | | | |
| --- | --- | --- | --- | --- |
|  | **Female** | | **Male** | |
|  | **Material deprivation** | **Social deprivation** | **Material deprivation** | **Social deprivation** |
| **10-14 years** | | | | |
| **Q2** | **1.15 (1.02, 1.31)*** | 1.13 (0.99, 1.30) | 1.02 (0.80, 1.31) | 1.00 (0.78, 1.29) |
| **Q3** | **1.21 (1.07, 1.38)*** | 1.14 (1.00, 1.31) | 1.13 (0.88, 1.44) | 1.01 (0.79, 1.30) |
| **Q4** | 1.09 (0.96, 1.23) | **1.16 (1.02, 1.33)*** | 0.83 (0.65, 1.07) | 0.99 (0.77, 1.28) |
| **Q5** | **1.54 (1.36, 1.75)*** | **1.38 (1.21, 1.58)*** | 1.05 (0.82, 1.34) | 1.18 (0.92, 1.53) |
| **15-19 years** | | | | |
| **Q2** | 1.09 (0.98, 1.22) | **1.21 (1.11, 1.31)*** | 1.08 (0.95, 1.23) | **1.12 (1.00, 1.25)*** |
| **Q3** | 1.12 (1.00, 1.25) | **1.25 (1.15, 1.35)*** | 1.06 (0.93, 1.20) | **1.14 (1.02, 1.27)*** |
| **Q4** | 1.06 (0.95, 1.19) | **1.38 (1.27, 1.50)*** | 1.00 (0.88, 1.13) | **1.23 (1.10, 1.37)*** |
| **Q5** | **1.23 (1.10, 1.37)*** | **1.69 (1.56, 1.84)*** | 1.11 (0.97, 1.26) | **1.53 (1.37, 1.71)*** |
| **20-24 years** | | | | |
| **Q2** | **1.25 (1.11, 1.41)*** | **1.13 (1.01, 1.28)*** | **1.22 (1.09, 1.36)*** | 1.00 (0.87, 1.15) |
| **Q3** | **1.27 (1.13, 1.43)*** | 1.10 (0.97, 1.23) | **1.50 (1.34, 1.67)*** | 1.05 (0.92, 1.20) |
| **Q4** | **1.22 (1.08, 1.37)*** | **1.25 (1.11, 1.41)*** | **1.37 (1.23, 1.53)*** | 1.04 (0.90, 1.19) |
| **Q5** | **1.52 (1.35, 1.71)*** | **1.45 (1.29, 1.64)*** | **1.78 (1.60, 1.99)*** | **1.23 (1.07, 1.41)*** |
| **25-44 years** | | | | |
| **Q2** | **1.30 (1.14, 1.48)*** | 1.13 (1.00, 1.28) | **1.25 (1.11, 1.41)*** | 1.07 (0.93, 1.22) |
| **Q3** | **1.61 (1.42, 1.84)*** | 1.13 (1.00, 1.28) | **1.59 (1.42, 1.79)*** | 1.12 (0.98, 1.28) |
| **Q4** | **1.63 (1.43, 1.85)*** | **1.41 (1.25, 1.60)*** | **1.57 (1.40, 1.77)*** | **1.29 (1.13, 1.47)*** |
| **Q5** | **2.04 (1.79, 2.33)*** | **1.99 (1.75, 2.25)*** | **2.11 (1.88, 2.37)*** | **1.77 (1.55, 2.02)*** |
| **45-64 years** | | | | |
| **Q2** | **1.26 (1.06, 1.49)*** | **1.15 (1.03, 1.27)*** | 1.15 (0.95, 1.39) | **1.19 (1.07, 1.34)*** |
| **Q3** | **1.35 (1.14, 1.59)*** | **1.26 (1.13, 1.40)*** | **1.39 (1.15, 1.68)*** | **1.23 (1.10, 1.38)*** |
| **Q4** | **1.41 (1.19, 1.67)*** | **1.52 (1.36, 1.69)*** | **1.52 (1.26, 1.83)*** | **1.61 (1.44, 1.80)*** |
| **Q5** | **1.71 (1.44, 2.02)*** | **2.49 (2.24, 2.77)*** | **1.66 (1.38, 2.01)*** | **2.77 (2.47, 3.11)*** |
| **65 years and over** | | | | |
| **Q2** | 1.10 (0.95, 1.26) | 1.01 (0.90, 1.15) | 1.06 (0.89, 1.26) | 0.88 (0.77, 1.01) |
| **Q3** | 1.01 (0.87, 1.16) | 0.91 (0.80, 1.03) | 1.17 (0.98, 1.39) | 0.97 (0.84, 1.11) |
| **Q4** | 1.06 (0.92, 1.23) | 0.95 (0.84, 1.08) | **1.23 (1.03, 1.46)*** | **1.26 (1.10, 1.44)*** |
| **Q5** | 0.91 (0.79, 1.05) | **1.41 (1.24, 1.59)*** | 1.18 (0.99, 1.41) | **1.71 (1.50, 1.96)*** |

Abbreviation: Q1-Q5: quintiles of deprivation index (Q1: least deprived area; Q5: most deprived area); CI: confidence interval

Note: Rate ratios adjusted for pandemic period. Statistically significant rate ratios are bolded (p-value < 0.05).

Table S2 Rate ratio of self-harm hospitalizations pre- and during COVID-19 pandemic across the quintile of material and social deprivation using Q1, the least deprived area, as reference, overall and by sex and age group, fiscal years 2015 – 2021, Canada excluding Quebec

| **Sex** | **Quantile** | **Unadjusted rate ratio (95% CI)** | | | |
| --- | --- | --- | --- | --- | --- |
|  |  | **Material deprivation** | | **Social deprivation** | |
|  |  | **Pre-pandemic** | **During pandemic** | **Pre-pandemic** | **During pandemic** |
| **Overall** | **Q2** | 1.16 (0.97, 1.39) | 1.14 (0.87, 1.50) | 1.13 (0.95, 1.35) | 1.13 (0.86, 1.47) |
|  | **Q3** | **1.25 (1.04, 1.49)*** | 1.21 (0.93, 1.59) | 1.15 (0.97, 1.38) | 1.14 (0.88, 1.49) |
|  | **Q4** | **1.21 (1.01, 1.45)*** | 1.17 (0.89, 1.53) | **1.30 (1.09, 1.55)*** | 1.30 (1.00, 1.70) |
|  | **Q5** | **1.46 (1.22, 1.74)*** | **1.36 (1.04, 1.78)*** | **1.70 (1.43, 2.03)*** | **1.68 (1.29, 2.19)*** |
| **Female** | **Q2** | 1.16 (0.96, 1.42) | 1.17 (0.86, 1.59) | 1.16 (0.95, 1.41) | 1.17 (0.87, 1.59) |
|  | **Q3** | **1.23 (1.01, 1.49)*** | 1.19 (0.88, 1.62) | 1.16 (0.96, 1.41) | 1.20 (0.89, 1.63) |
|  | **Q4** | 1.18 (0.97, 1.44) | 1.17 (0.86, 1.59) | **1.32 (1.08, 1.60)*** | 1.34 (0.99, 1.82) |
|  | **Q5** | **1.43 (1.18, 1.74)*** | 1.34 (0.99, 1.83) | **1.67 (1.38, 2.03)*** | **1.71 (1.27, 2.32)*** |
| **Male** | **Q2** | 1.18 (0.98, 1.43) | 1.10 (0.83, 1.46) | 1.06 (0.88, 1.27) | 1.03 (0.77, 1.36) |
|  | **Q3** | **1.31 (1.08, 1.58)*** | 1.31 (0.98, 1.74) | 1.12 (0.93, 1.35) | 1.02 (0.77, 1.35) |
|  | **Q4** | **1.31 (1.08, 1.58)*** | 1.22 (0.92, 1.62) | **1.22 (1.01, 1.48)*** | 1.19 (0.89, 1.57) |
|  | **Q5** | **1.54 (1.28, 1.87)*** | **1.45 (1.09, 1.93)*** | **1.69 (1.40, 2.04)*** | **1.53 (1.15, 2.03)*** |

Abbreviation: Q1-Q5: quintiles of deprivation index (Q1: least deprived area; Q5: most deprived area); CI: confidence interval

Note: Statistically significant rate ratios are bolded (p-value < 0.05).

Table S3 Rate ratio of self-harm hospitalizations pre- and during COVID-19 pandemic across the quintile of material and social deprivation using Q1, the least deprived area, as reference, overall and by sex and age group, fiscal years 2015 – 2021, Canada excluding Quebec

| **Quintile** | **Unadjusted rate ratio (95% CI)** | | | | | | | | |
| --- | --- | --- | --- | --- | --- | --- | --- | --- | --- |
|  | **Female** | | | | **Male** | | | | |
|  | **Material deprivation** | | **Social deprivation** | | **Material deprivation** | | | **Social deprivation** | |
|  | **Pre-pandemic** | **During pandemic** | **Pre-pandemic** | **During pandemic** | **Pre-pandemic** | **During pandemic** | **Pre-pandemic** | | **During pandemic** |
| **0-14 years** | | | | | | | | | |
| **Q2** | **1.17 (1.01, 1.35)** | 1.11 (0.88, 1.40) | 1.12 (0.95, 1.32) | 1.17 (0.93, 1.45) | 0.99 (0.74, 1.34) | 1.07 (0.70, 1.64) | 0.94 (0.70, 1.25) | | 1.17 (0.74, 1.86) |
| **Q3** | **1.24 (1.07, 1.44)** | 1.15 (0.92, 1.45) | 1.11 (0.94, 1.31) | 1.23 (0.99, 1.54) | 1.18 (0.89, 1.58) | 1.02 (0.67, 1.56) | 1.08 (0.80, 1.45) | | 0.83 (0.52, 1.33) |
| **Q4** | 1.11 (0.96, 1.28) | 1.03 (0.82, 1.30) | 1.15 (0.97, 1.36) | 1.19 (0.96, 1.49) | 0.98 (0.73, 1.32) | **0.51 (0.33, 0.80)** | 0.97 (0.73, 1.31) | | 1.04 (0.65, 1.65) |
| **Q5** | **1.71 (1.48, 1.98)** | 1.16 (0.92, 1.45) | **1.41 (1.19, 1.66)** | **1.32 (1.06, 1.64)** | 1.18 (0.88, 1.58) | 0.78 (0.51, 1.20) | 1.16 (0.86, 1.57) | | 1.24 (0.77, 1.99) |
| **15-19 years** | | | | | | | | | |
| **Q2** | 1.10 (0.96, 1.25) | 1.09 (0.88, 1.35) | **1.23 (1.11, 1.35)** | 1.17 (1.00, 1.37) | 1.09 (0.93, 1.27) | 1.07 (0.85, 1.35) | 1.11 (0.98, 1.27) | | 1.14 (0.92, 1.42) |
| **Q3** | 1.12 (0.98, 1.27) | 1.12 (0.90, 1.38) | **1.24 (1.13, 1.37)** | **1.25 (1.07, 1.47)** | 1.07 (0.92, 1.25) | 1.03 (0.82, 1.30) | **1.14 (1.00, 1.30)** | | 1.12 (0.90, 1.39) |
| **Q4** | 1.08 (0.94, 1.22) | 1.04 (0.84, 1.28) | **1.37 (1.24, 1.51)** | **1.40 (1.19, 1.64)** | 1.05 (0.90, 1.22) | 0.87 (0.69, 1.09) | **1.21 (1.07, 1.38)** | | **1.27 (1.02, 1.58)** |
| **Q5** | **1.26 (1.11, 1.44)** | 1.14 (0.92, 1.41) | **1.70 (1.54, 1.88)** | **1.67 (1.43, 1.96)** | 1.16 (1.00, 1.35) | 0.98 (0.78, 1.23) | **1.57 (1.38, 1.79)** | | **1.41 (1.14, 1.75)** |
| **20-24 years** | | | | | | | | | |
| **Q2** | **1.24 (1.07, 1.43)** | **1.30 (1.06, 1.59)** | 1.10 (0.95, 1.27) | **1.22 (1.00, 1.48)** | **1.29 (1.13, 1.48)** | 1.05 (0.87, 1.26) | 1.03 (0.88, 1.21) | | 0.93 (0.72, 1.20) |
| **Q3** | **1.28 (1.11, 1.48)** | **1.25 (1.02, 1.54)** | 1.08 (0.94, 1.25) | 1.13 (0.93, 1.37) | **1.51 (1.32, 1.72)** | **1.47 (1.23, 1.77)** | 1.09 (0.93, 1.28) | | 0.96 (0.75, 1.24) |
| **Q4** | **1.18 (1.02, 1.36)** | **1.32 (1.08, 1.62)** | **1.24 (1.07, 1.43)** | **1.28 (1.05, 1.56)** | **1.37 (1.20, 1.57)** | **1.36 (1.13, 1.64)** | 1.06 (0.90, 1.25) | | 0.98 (0.76, 1.26) |
| **Q5** | **1.48 (1.28, 1.71)** | **1.64 (1.34, 2.00)** | **1.39 (1.20, 1.60)** | **1.64 (1.35, 1.99)** | **1.80 (1.58, 2.06)** | **1.73 (1.44, 2.08)** | **1.29 (1.10, 1.51)** | | 1.10 (0.86, 1.42) |
| **25-44 years** | | | | | | | | | |
| **Q2** | **1.26 (1.09, 1.47)** | **1.38 (1.06, 1.79)** | 1.13 (0.98, 1.32) | 1.12 (0.89, 1.40) | **1.29 (1.12, 1.49)** | 1.16 (0.95, 1.40) | 1.09 (0.93, 1.28) | | 1.02 (0.81, 1.28) |
| **Q3** | **1.65 (1.42, 1.92)** | **1.52 (1.17, 1.97)** | 1.14 (0.98, 1.32) | 1.12 (0.89, 1.40) | **1.59 (1.37, 1.83)** | **1.60 (1.32, 1.94)** | 1.13 (0.96, 1.33) | | 1.11 (0.88, 1.39) |
| **Q4** | **1.61 (1.39, 1.87)** | **1.66 (1.28, 2.16)** | **1.43 (1.24, 1.67)** | **1.36 (1.09, 1.71)** | **1.66 (1.44, 1.91)** | **1.38 (1.14, 1.67)** | **1.29 (1.09, 1.51)** | | **1.28 (1.02, 1.61)** |
| **Q5** | **2.07 (1.79, 2.40)** | **1.96 (1.51, 2.55)** | **1.95 (1.68, 2.27)** | **2.07 (1.66, 2.60)** | **2.17 (1.89, 2.51)** | **1.95 (1.61, 2.37)** | **1.82 (1.54, 2.14)** | | **1.65 (1.31, 2.07)** |
| **45-64 years** | | | | | | | | | |
| **Q2** | **1.26 (1.03, 1.53)** | 1.26 (0.92, 1.74) | 1.08 (0.95, 1.22) | **1.34 (1.11, 1.60)** | 1.16 (0.92, 1.45) | 1.13 (0.80, 1.60) | **1.18 (1.03, 1.35)** | | 1.23 (0.99, 1.52) |
| **Q3** | **1.37 (1.12, 1.67)** | 1.30 (0.94, 1.79) | **1.21 (1.07, 1.38)** | **1.40 (1.16, 1.68)** | **1.36 (1.09, 1.70)** | **1.46 (1.03, 2.07)** | **1.25 (1.09, 1.43)** | | 1.18 (0.95, 1.46) |
| **Q4** | **1.46 (1.20, 1.78)** | 1.30 (0.94, 1.79) | **1.46 (1.29, 1.66)** | **1.66 (1.39, 2.00)** | **1.50 (1.20, 1.87)** | **1.57 (1.11, 2.22)** | **1.65 (1.45, 1.89)** | | **1.50 (1.22, 1.86)** |
| **Q5** | **1.72 (1.41, 2.09)** | **1.67 (1.21, 2.31)** | **2.40 (2.11, 2.72)** | **2.76 (2.30, 3.32)** | **1.70 (1.36, 2.12)** | **1.57 (1.11, 2.23)** | **2.80 (2.45, 3.20)** | | **2.72 (2.19, 3.36)** |
| **65 years and over** | | | | | | | | | |
| **Q2** | 1.13 (0.95, 1.35) | 1.01 (0.80, 1.27) | 1.06 (0.91, 1.23) | 0.90 (0.74, 1.11) | 1.02 (0.83, 1.25) | 1.16 (0.83, 1.62) | 0.90 (0.77, 1.05) | | 0.85 (0.66, 1.09) |
| **Q3** | 0.99 (0.83, 1.18) | 1.05 (0.84, 1.32) | 0.90 (0.77, 1.05) | 0.93 (0.76, 1.15) | 1.18 (0.96, 1.44) | 1.14 (0.82, 1.59) | 1.10 (0.94, 1.28) | | **0.69 (0.53, 0.89)** |
| **Q4** | 1.08 (0.90, 1.28) | 1.03 (0.82, 1.30) | 0.93 (0.80, 1.09) | 1.00 (0.82, 1.23) | 1.21 (0.99, 1.48) | 1.29 (0.93, 1.80) | **1.32 (1.13, 1.53)** | | 1.14 (0.88, 1.47) |
| **Q5** | 0.92 (0.77, 1.09) | 0.90 (0.72, 1.14) | **1.46 (1.25, 1.69)** | **1.29 (1.05, 1.58)** | 1.10 (0.90, 1.34) | **1.42 (1.01, 1.97)** | **1.79 (1.54, 2.09)** | | **1.54 (1.20, 1.99)** |

Abbreviation: Q1-Q5: quintiles of deprivation index (Q1: least deprived area; Q5: most deprived area); CI: confidence interval

Note: Statistically significant rate ratios are bolded (p-value < 0.05).
